# Determinants of COVID-19 Vaccine Uptake Among Health Workers and General Public in Tanzania

**DOI:** 10.24248/eahrj.v8i1.760

**Published:** 2024-03-28

**Authors:** Kijakazi Obed Mashoto, Mukome A. Nyamhagatta, Maro Mwikwabe Chacha, Pricillah Kinyunyi, Ismail Habib, Masanja Robert Kasanzu, Florian Tinuga

**Affiliations:** a National Institute for Medical Research, Dar es Salaam, Tanzania; b Ministry of Health, Dodoma, Tanzania

## Abstract

**Background:**

Insufficient knowledge about COVID-19 and low socioeconomic status have been associated with distrustful attitudes towards vaccination against COVID-19.

**Objective:**

The aim of this study was to explore determinants of COVID-19 vaccine uptake among the general population and health workers.

**Methods:**

A cross sectional study was conducted in 16 councils which included; Milele, Mpanda, Newala, Simanjiro, Nanyumbu, Muleba, Longido, Ulanga, Igunga, Mbulu, Karatu, Mufindi, Mvomero, Kilolo and Tabora Town. A total of 427 health care workers and 1,907 individuals were sampled from health facilities and households. Structured questionnaires were used to collect the required information.

**Results:**

Although the majority (93.2%) of health workers were vaccinated, 35.4% perceived their risk of getting COVID-19 infection as high. Self-reported uptake of COVID-19 vaccine was 42.4% among the general population. Significantly low proportion of the general population in Mufindi district council (7.5%) were vaccinated against COVID-19. Health workers' knowledge and perception on COVID-19 vaccination did not vary with socio-demographic factors. Among the general population, those who were separated/divorced (ARR: 0.8: 95% CI; 0.7 to 0.9), those who attained primary level of education (ARR: 0.8: 95% CI; 0.7 to 0.9), self-employed (ARR: 0.8: 95% CI; 0.7 to 0.9) and unemployed (ARR: 0.7: 95% CI; 0.6 to 0.8) were less likely to be vaccinated against COVID-19. Having positive attitude (ARR: 1.2: 95% CI; 1.1 to 1.5) and perception (ARR:1.8: 95% CI; 1.5 to 2.2), and knowledge on COVID-19 prevention (ARR: 3.0: 95% CI; 2.1to 4.4) increased the likelihood COVID-19 vaccine uptake. Prior experience of vaccination against other diseases (ARR:1.2: 95% CI; 1.0 to1.3), having history of chronic diseases (ARR:1.3: 95% CI; 1.2 to 1.4) and a family member who died of COVID-19 (ARR:1.3: 95% CI; 1.1to1.4) were also determinants of COVID-19 vaccine uptake.

**Conclusion:**

Uptake of COVID-19 vaccine among the general population was significantly low among individuals with primary level of education, self-employed, unemployed, and those who were divorced or separated. Individuals with comprehensive knowledge on COVID-19 vaccination, those with positive attitude and perception on COVID-19 vaccination, having history of chronic diseases, prior vaccination against other diseases, and having a family member who succumbed to COVID-19 increased the likelihood COVID-19 vaccine uptake among the general population. Provision of health education and implementation of socio-behavioural communication change interventions are necessary to equip the general population with appropriate knowledge to transform their negative attitude and perception on COVID-19 vaccination.

## BACKGROUND

Coronavirus disease (COVID-19) is an infectious condition caused by the severe acute respiratory syndrome corona virus type 2 (SARS-CoV-2) virus. It poses a higher risk of severe illness in older people and those with underlying medical conditions such as; cardiovascular disease, diabetes, chronic respiratory disease, or cancer. The virus spreads through small liquid particles expelled from the mouth or nose of an infected person when they cough, sneeze, speak, sing or breathe. These particles range from larger respiratory droplets to smaller aerosols.^1^

For decades, vaccinations have been considered the most effective method for control of rapidly spreading infectious diseases. To achieve the necessary herd immunity to control viral transmission and stop the pandemic, vaccinating more than 70% of the population is crucial and requires strong acceptance and low hesitation levels throughout the population.^2^ As COVID-19 vaccines are rolled out globally, vaccine hesitancy has stimulated public debates across social media and various multimedia platforms. The level of vaccine hesitancy and contributing factors significantly vary based on individual's location, background, and community.^3,4^ Being cognisant of factors contributing to vaccine hesitancy may help in developing strategies for delivering vaccine to the most vulnerable populations. Insufficient knowledge, negative attitudes and perceptions about COVID-19 vaccines contribute to vaccine hesitancy which lead to low immunisation coverage in most countries.^4,5^ Studies conducted around the world have indicated that knowledge, attitude and perception significantly influence uptake of COVID-19 vaccination.^6–8^ Scepticism towards vaccination is associated with minority ethnicity, lower levels of education, lower income, poor knowledge of COVID-19, and non-compliance with government COVID-19 guidelines.^9–13^ However, synthesis of data from 5 large scale surveys conducted in 22 countries across Southern, Eastern, Central and Western Africa showed that demographic factors have no clear impact on vaccine acceptance, although variations exist within country regions.^14,15^

Tanzania welcomed its first shipment of over one million doses of Johnson & Johnson COVID-19 vaccines in July 2021 from the United States as part of the COVAX arrangement. The first phase of the COVID-19 vaccination rollout which began in August 2021, prioritised frontline health workers, elderly (individuals aged 50 years and above) and those with chronic illnesses. Currently, 5 types of vaccines have been approved, received and distributed across the nation. Study conducted by Msuya et al^16^ highlighted a low vaccine uptake rate (18%) in Tanzania. However, the selection of the study sites was based on vaccine wastage rates might have biased the uptake estimates and undermined the factors which associate with vaccine uptake or hesitancy. Thus, this study employs a multistage random sampling of regions and councils to identify factors associated with varying levels of COVID-19 vaccine uptake among the general population and health workers.

## METHODS

### Study Design, Site and Population

A cross sectional study was conducted in 16 district and municipal councils in 8 regions to investigate the factors influencing COVID-19 vaccine uptake among the general population and health workers in Tanzania. The district and municipal councils which participated in this study included; Milele, Mpanda, Newala, Simanjiro, Nanyumbu, Muleba, Longido, Ulanga, Igunga, Mbulu, Karatu, Mufindi, Mvomero, Kilolo and Tabora Town.

### Sample Size

Assuming that 50% of health workers possess comprehensive knowledge about COVID-19 vaccination, with a confidence level of 95% and a margin of error of 5%, the initial sample size calculated for healthcare providers was 384. Accounting for non-response of 15%, the sample was adjusted to 442. Based on the fact that 52.2% of the Tanzanian population possess good knowledge of COVID-19 vaccines^17^ and that 48.3% had inadequate knowledge on the causes of COVID-19,^18^ a decision was made to assume that 50% of the general population have some knowledge on COVID-19 vaccination. Setting the confidence level at 95% and a margin of error at 2.3%, the calculated sample size for the general population was 1,812. Considering a non-response of 10%, the sample was increased to 1,993.

### Sampling Procedures

In Tanzania, there are 8 Health Sector Zones and 7 Administrative Zones. The study was about health issues, deemed appropriate to use Health Sector Zones. So, in each Health Sector Zone one region was randomly selected, comprising Arusha, Iringa, Kagera, Katavi, Manyara, Morogoro, Mtwara and Tabora. In each selected region, 2 councils were randomly selected.

Stratified by rural-urban classification, 1 ward was randomly selected from each stratum, giving a total of 32 wards. In each ward, four (4) villages/streets were randomly selected, leading to the sampling of 128 villages and streets for the entire study. In each selected village or street, 15 households were selected using simple random sampling techniques. In each selected household, one respondent aged 18 years or above was sampled for the interview. A total of 1,907 individuals were interviewed.

Eight region hospitals and 16 District Hospitals were purposefully sampled in the selected regions and councils respectively. Thirty-two (32) health centres and 64 dispensaries were randomly selected from respective lists of health centres and dispensaries in the councils. This resulted to a total of 120 health facilities participating in this study. Within each participating hospital, 6 health workers were sampled, and in each health centre, 4 health workers were sampled. At the dispensary level, up to 3 health workers were interviewed during each visit. Thus, in total 427 health workers were interviewed.

In the context of this study health workers included doctors, nurses, pharmacists, lobotomy technologists and technicians, health or medical attendants and all other individuals working at the health facilities.

### Data Collection

A structured questionnaire was used to collect the required information from the study population. Initially crafted in English, the questionnaire was subsequently translated into Kiswahili. To ensure preservation of the meaning, the questions were back translated into English by an independent person.

Prior to commencing field work, all instruments were pre-tested in the non-survey site. Adjustments were made as necessary based on the findings of the pre-test. Data collection was carried out by trained Research Assistants (RAs) using Open Data Kit (ODK).

### Assessment of Knowledge, Attitude and Perception on COVID-19 Vaccination

Knowledge about COVID-19 vaccination was assessed by 9 items (Table 1), each with 2 options, “yes” coded as 1 and “no” coded as 2. Respondents were requested to respond to statements which measured their knowledge on benefits of COVID-19 vaccination, delivery mode, side effects, importance of combining vaccination with other preventive measures and which population is targeted for vaccination. Knowledge score was computed by summing up the score for each item.^19^ Since there was one false statement, response codes were reversed before calculating the total knowledge score. Using the mean score, the overall knowledge score was dichotomised into comprehensive knowledge and limited knowledge.

**TABLE 1:** Questions and Statements Used to Assess Knowledge, Attitude and Perception on COVID-19 Vaccination

COVID-19 vaccination knowledge (9 items)	Attitude towards COVID-19 vaccination (8 items)	Perception on COVID-19 vaccination (10 items)
Do you know that COVID-19 Vaccine may protect you from adverse effects of the disease?	I will take the COVID-19 vaccine without any hesitation, if it is available at my neighbourhood	COVID-19 vaccines work
Are you aware that COVID-19 Vaccine is available free of charge at the health facilities in your community?	I will also encourage my family/friends/relatives to get vaccinated.	COVID-19 vaccines are safe for use in humans
Do you know that administration of COVID-19 vaccine may cause mild side effects?	It is not possible to reduce the incidence of COVID-19 without vaccination	COVID-19 vaccines have passed rigorous quality control for use
Can vaccinated person get infected with COVID-19?	The COVID-19 vaccine should be distributed fairly to all of us.	Benefits of COVID-19 vaccine outweighs the risks.
vaccine can be given to people who had been infected/sick with COVID-19?	If everyone in the society maintains the preventive measures, the COVID-19 pandemic can be eradicated without vaccination	Safety of COVID-19 vaccine is compromised by shorten time taken to develop and approve it
COVID-19 K13. A person who has received COVID-19 vaccine needs to continue following other preventive methods (e.g. distancing, hand washing, use of mask)	It is essential to vaccinate a large proportion of the population in order to defeat COVID-19 pandemic	The Ministry of Health has ensured that available vaccines in Tanzania are safe
COVID-19 vaccines can reduce the risk/chance of being infected with COVID-19?	COVID-19 vaccination can cause COVID-19 infections to a person	Fear of serious adverse events prevents me from taking COVID-19 vaccines
COVID-19 vaccines prevent severe infection?	COVID-19 vaccine provide a long-term protection against COVID-19 infection	Fear of side effects prevent me from taking COVID-19 vaccines
COVID-19 vaccines reduce deaths due to COVID-19?		Do not fully support the government move for vaccinating people against COVID-19 The COVID-19 vaccines is essential for us

Attitude was assessed by 8 items (Table 1). The assessment focused on the respondent's assertiveness on willingness to be vaccinated or to encourage family member to be vaccinated, effect of COVID-19 on disease prevention, importance of vaccination and adherence to other preventive measures. A 5-point Likert scale was used to measure the extent to which respondent agreed or disagreed with each statement. If respondent strongly agreed with the statement a score of 1 was assigned to the respective statement. A score of 5 was given to the statement which respondent strongly disagree with. Two out of 8 were negative statements and therefore, their codes were reversed prior to computation of the attitude score. The attitude score was constructed by summing up the score for each item, and the mean score was used to dichotomise the overall score into positive and negative attitudes towards COVID-19 vaccination.^19^

Perception of COVID-19 vaccination focused on the use of the vaccine, trust in the precautionary measures implemented by the Ministry of Health to safeguard Tanzanians' health and concerns over the effectiveness and safety of the vaccine. The assessment of COVID-19 vaccination perception comprised of 10 items (Table 1). A 5-point Likert scale was used to measure the extent to which respondent agreed or disagreed with each statement. If respondent strongly agreed with the statement, a score of 1 was assigned to the respective statement. A score of 5 was given to the statement which respondent strongly disagreed with. The final score was obtained by summing up individual item scores, and the total score was dichotomised into positive and negative attitude using the Mean score as the cut-off point.^20^

To assess uptake of COVID-19 vaccine, respondents were asked if they have received COVID-19 vaccine. The response options were yes (coded 1) and no (coded 2).

### Data Analysis

The collected independent variables included; social demographic characteristics, knowledge, perception and attitude towards COVID-19 vaccination. Uptake of COVID-19 vaccine was the dependent variable assessed. The collected quantitative data was processed in Statistical Software for Data Science (STATA) version 18.^21^

Data analysis involved describing the demographic characteristics of the participants and summarising categorical variables using percentages, along with mean and standard deviation for normally distributed data. Chi-squared analysis was used to compare proportions of individuals with knowledge, positive attitude and perception on COVID-19 vaccination against their counterpart groups.

For variable measured using the Likert scale, response options were re-categorised into agree or disagree. Scores for knowledge, attitude and perception towards COVID-19 vaccination were constructed, and then categorised into high and low knowledge, as well as positive and negative attitude and perception.

Principle Components Analysis (PCA) was performed to establish factor scores or domain variable for KAP, which were subsequently used in logistic regression analysis to explore the effects of KAP on COVID-19 vaccines uptake. Finally, logistic regression analysis was performed to identify factors which influencing the uptake of COVID-19 vaccines in Tanzania.

### Ethical Considerations

This study was approved by the National Health Research Ethics Committee of Tanzania (Certificate reference number NIMR/HQ/R.8a/Vol.IX/3981). Study participants provided written informed consent before being enrolled for the study.

## RESULTS

### Demographics of Studied General Population and Health Workers

**FIGURE 1:**
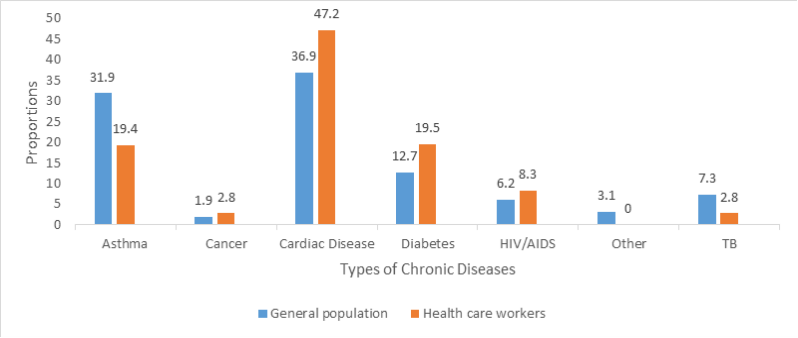
Proportion of Respondents with Chronic Diseases

The response rate for the general population and Healthcare Workers (HWs) was 95.7% and 96.6% respectively. For both groups (general population and HWs), majority of respondents were aged between 18 and 49 years, married and residing in urbans (Table 2). Of the interviewed individuals 13.6% of the general population and 8.4% of HWs had history of chronic illnesses. Notably, a significantly high proportion of HWs (47.2%) and the general population (36.9%) had a history of cardiac disease (Figure 1).

**TABLE 2:** Socio-demographics of Respondents

Variables	General Population (N = 1907) n (%)	Health Workers (N = 427) n (%)
Age group (Years)		
18–29	512 (26.7)	125 (29.3)
30–39	574 (29.9)	189 (44.3)
40–49	421 (21.9)	66 (15.5)
50–59	250 (13.0)	44 (10.3)
60+	163 (8.5)	3 (0.7)
Sex		
Female	1,034 (54.2)	242 (56.7)
Male	873 (45.8)	185 (43.3)
Marital status		
Cohabiting	80 (4.2)	4 (0.9)
Divorced/separated	107 (5.6)	8 (1.9)
Married	1,300 (68.2)	290 (67.9)
Single	420 (22.0)	125 (29.3)
Level of education		
College/University	116 (6.1)	-
No formal education	191 (10.0)	-
Primary education	1,177 (61.7)	-
Secondary Education	423 (22.2)	-
Employment status		NA
Government sector	79 (4.1)	422
Housewife	324 (17.0)	
Private Sector	110 (5.8)	
Self employed	1,035 (54.3)	
Student	38 (2.0)	
Unemployed	321 (16.8)	
Place of residence		
Rural	715 (37.5)	146 (34.2)
Urban	1,192 (62.5)	281 (65.8)

Given the nature of their job, almost all HWs (99.1%) believed that COVID-19 is present in Tanzania. Additionally, nearly two thirds reported administering COVID-19 vaccine to clients, but only 50% had received training on COVID-19 vaccine, and 35.4% believed that they were at high risk of contracting a COVID-19 infection (Table 3).

**TABLE 3:** Family Member COVID-19 Experience and Mortality and Vaccine Uptake

Variables	General Population (N = 1907) n (%)	Health Workers (N = 427) n (%)
Family member infected with COVID-19	132 (6.9)	7 (18.1)
Family member died from COVID-19	24 (18.2)	25 (32.5)
Received COVID-19 vaccine	809 (42.4)	394 (92.3)
Ready for vaccine booster dose	732 (90.5)	367 (93.1)
Ever received other vaccine	1,517 (79.5)	417 (97.7)
Agreed COVID-19 is present	1725 (90.5)	423 (99.1)

### Corona Virus Infection and COVID-19 Vaccine Uptake

A significantly higher percentage of HWs (18%) than the general population (6.9%) acknowledged to have had family members infected with COVID-19. Among those with infected family members, 32.5% of HWs and 18.2% of general population affirmed that the infected members had passed away (Table 3).

Majority of the general population (79.5%) and HWs (97.7%) reported having been vaccinated against other diseases and were ready for booster doses. However, compared to the general population (42.4%) a significantly high proportion of HWs (92.3%) claimed to have been vaccinated against COVID-19 (Table 3). Notably, 92.7% of HWs who believed that COVID-19 is present in the country were vaccinated against the disease, whereas only 50% of those who did not believe in the presence of the disease is in Tanzania received the vaccine.

**FIGURE 2:**
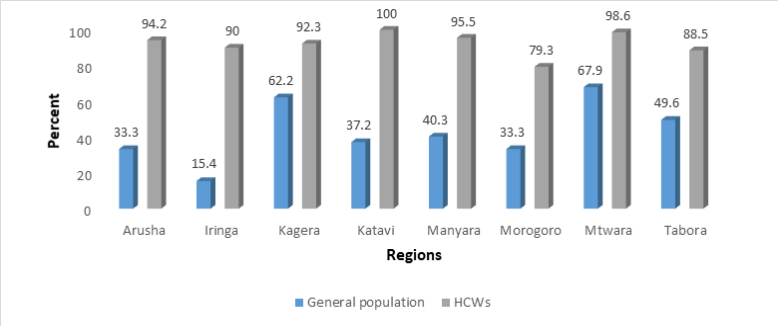
Uptake of COVID-19 Vaccine among Health Workers and General Population by Region

**FIGURE 3:**
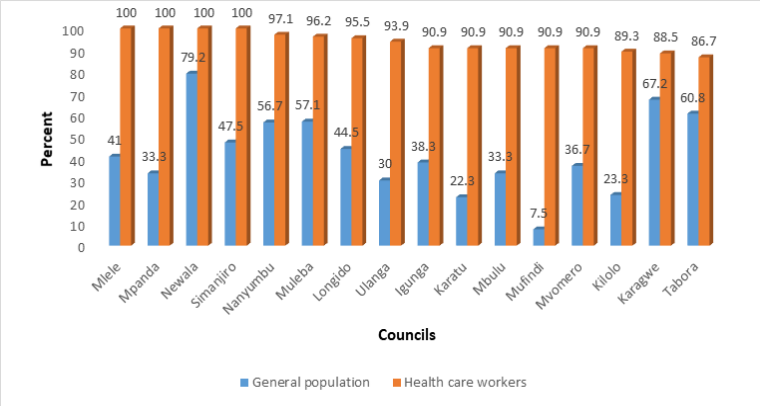
Uptake of COVID-19 Vaccine among Health Workers and General Population by Council

There is regional and district variation in the uptake of the COVID-19 vaccine, particularly among the general population. HWs in Morogoro demonstrated a lower uptake of COVID-19 vaccine compared to other regions (Figure 2). Self-reported uptake of COVID-19 vaccine was below 42% in half of the studied district councils, with only 7.5% of the general population in Mufundi reporting to have been vaccinated against COVID-19. Newala, Karagwe and Tabora district councils seemed to have good COVID-19 vaccine coverage compared to other studied district councils (Figure 3).

### Knowledge on COVID-19 Vaccination

In general, HWs exhibited good knowledge regarding COVID-19 vaccine benefits and its side effects. The majority (94.6%) of HWs were aware that the administration of the COVID-19 vaccine may cause mild size effects.

In contrast, 54.9% of the general population possessed this knowledge. Additionally, slightly over half of the general population (53.4%) was unaware that a person who had received COVID-19 vaccine could still get infected with COVID-19 (Table 4). There was a significantly lower proportion of the general population (78.4%) compared to HWs (99.5%), who agreed that it is crucial to continue with other preventive methods even after receiving COVID-19 vaccine.

**TABLE 4:** Health Workers' and General Population's Knowledge on COVID-19 Vaccination

Knowledge Statement	General Population (N = 1907)	Health Workers (N = 427)
Administration of COVID-19 vaccine may cause mild side effects	1,133 (59.4)	404 (94.6)
Vaccinated person get infected with COVID-19	1,018 (53.4)	390 (91.3)
COVID-19 vaccine administration to people infected with COVID-19	1,040 (54.5)	371 (86.9)
Continuing with other preventive methods after receiving COVID-19 vaccine	1,496 (78.4)	425 (99.5)
COVID-19 vaccines reduce the risk of being infected	1,387 (72.7)	326 (76.3)
COVID-19 vaccines reduce deaths due to COVID-19	1,427 (74.8)	422 (98.8)
COVID-19 vaccines prevent severe infections	1371 (71.9)	420 (98.4)

### Attitude Towards COVID-19 Vaccines

More than half of HWs (56.4%) and 41.1% of the general population believed that it is not possible to reduce the incidence of COVID-19 without vaccination. Furthermore, majority of both HWs and the general population agreed that COVID-19 vaccines should be fairly distributed to all. However, it is important to note that 57.9% of the general population and 83% of HWs believed that if everyone in the society adheres to and maintains preventive measures, the COVID-19 pandemic can be eradicated without vaccination. Whereas 71% of the general population felt that it is essential to vaccinate a large proportion of the population in order to defeat the COVID-19 pandemic, less than half of the HWs (48.9%) shared this belief. Interestingly, a substantial proportion of respondents believed that vaccine can cause COVID-19 infection to a person (Table 5).

**TABLE 5:** Health Workers and General Population with Positive Attitude Towards COVID-19 Vaccination

Statement	General Population n (%)	Health Workers n (%)
It is not possible to reduce the incidence of COVID-19 without vaccination	783 (41.1)	241 (56.4)
The COVID-19 vaccine should be distributed fairly to all of us.	1392 (73.0)	354 (82.9)
If everyone in the society maintains the preventive measures, the COVID-19 pandemic can be eradicated without vaccination	1105 (57.9)	354 (82.9)
It is essential to vaccinate a large proportion of the population in order to defeat COVID-19 pandemic	1368 (71.7)	209 (48.9)
COVID-19 vaccination can cause COVID-19 infections to a person	501 (26.3)	147 (34.4)
COVID-19 vaccine provide a long-term protection against COVID-19 infection	1121 (58.8)	341 (79.9)

### Perception on COVID-19 Vaccination

HWs perceived COVID-19 vaccine as effective (98.6%) and safe for use (97.4%). On the other hand, the general population was doubtful. Slightly above 50% and 48.6% of the general population believed that the vaccine is effective and safe respectively. Furthermore, only 52.1% of the general population thought that the benefits of COVID-19 vaccine outweigh the risks. Compared with the HWs (15%), significantly high proportion of the general population (34.3%) hesitate to take up CIVID-19 vaccine because of its side effects. Only 56% of the general population was willing to recommend or encourage a friend or relative to take up COVID-19 vaccine (Table 6).

**TABLE 6:** Health Workers and General Population with Positive Perception on COVID-19 Vaccination

Perception statements	General Population N (%)	Health Workers N (%)
COVID-19 vaccines work	984 (51.6)	421 (98.6)
COVID-19 vaccines are safe for use in humans	927 (48.6)	415 (97.2)
Routine vaccines are safer than COVID-19 vaccine	734 (38.5)	183 (41.9)
COVID-19 vaccines have passed rigorous quality control for use	850 (44.60)	379 (88.76)
Benefits of COVID-19 vaccine outweighs the risks	994 (52.1)	415 (97.2)
Shortcuts which are taken to develop and approve vaccines during a pandemic emergency compromise their safety	436 (22.9)	108 (25.3)
I trust that the Ministry of Health has ensured that the vaccines made available in Tanzania are safe	1,037 (54.4)	414 (97.0)
My concerns about related to side effects (minor/short term effects) prevent me from taking COVID-19 vaccine	654 (34.3)	64 (15.0)
The government is pushing you into a vaccination decision you do not fully support	792 (41.5)	30 (7.0)
The COVID-19 vaccines is essential for us	1,097 (57.5)	420 (98.4)
I will take the COVID-19 vaccine without any hesitation, if it is available at my neighbourhood	1,020 (53.5)	403 (94.4)
I will also encourage my family/friends/relatives to get vaccinated.	1,068 (56.0)	424 (99.3)

**FIGURE 4:**
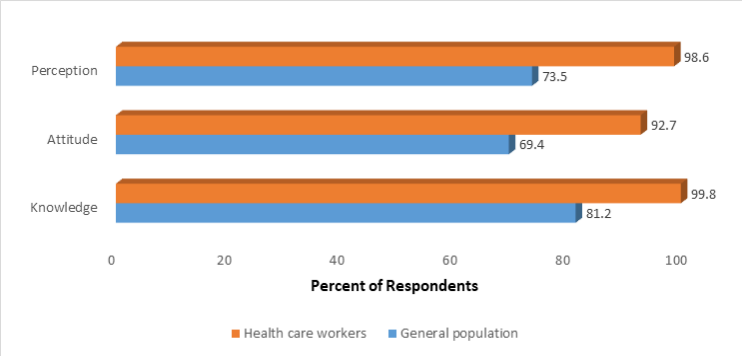
Percent of Health Workers and General Population with Adequate Knowledge, and Positive Attitude and Perception on COVID-19 Vaccination

In generally, HWs exhibited a positive attitude and perception towards COVID-19 vaccine, and demonstrated greater knowledge compared to the general population (Figure 4). However, HWs' knowledge and perception regarding COVID-19 vaccine did not vary with socio-demographic factors. The attitude of HWs towards COVID-19 vaccination, however, varied with region and vaccination status against COVID-19. In Morogoro (81%) and Mtwara (88%), a lower proportion of HWs exhibited a positive attitude towards COVID-19 vaccination, compared to other regions (over 90%). HWs who received the COVID-19 vaccine (93.9%) were more likely to have positive attitude towards COVID-19 vaccination than those who did not vaccinate (78.8%). Among the general population, factors such as; age, marital status, education, region, COVID-19 vaccination status, employment status, family members' COVID-19 status, vaccination against other diseases status, belief in the presence of COVID-19 in the country and perceived risk played a role. Individuals aged 60+ years, those cohabiting, divorced/separated, with no formal education, unemployed, without family member affected by COVID-19, not vaccinated against COVID-19 or other diseases, not believing that COVID-19 is present in the country, and those with perceived low risk of being infected with COVID-19 were more likely to have limited knowledge, negative attitude and perception towards COVID-19 vaccination. Whereas non-HWs respondents from Arusha, Manyara, Mtwara were less likely to have positive attitude and perception towards COVID-19 vaccination, those in Tabora were more likely to have limited knowledge on COVID-19 vaccination (Table 7).

**TABLE 7:** Knowledge, Attitude and Perception on COVID-19 Vaccine Among General Population by Sociodemographics

Variables	Adequate knowledge	Positive attitude	Positive perception
Age			
18–29	421 (82.2)	348 (67.9)	384 (75.0)
30–39	470 (81.9)	409 (71.2)	442 (77.0)
40–49	353 (83.8)	306 (72.7)	313 (74.3)
50–59	207 (82.8)	177 (70.8)	172 (68.80)
60+	97 (64.7)**	84 (56.0)**	90 (60.0)**
Marital status			
Cohabiting	53 (66.2)**	44 (55.0)**	53 (66.25)**
Divorced/separated	72 (67.3)**	65 (60.7)**	71 (66.36)**
Married	1081 (83.1)	917 (70.5)	962 (74.0)
Single	342 (81.4)	298 (70.9)	315 (75.0)
Vaccinated against COVID-19			
No	772 (70.3)**	614 (55.9)**	739 (67.3)**
Yes	776 (95.9)	710 (87.8)	662 (81.8)
Level of education			
College/University	112 (96.55)	102 (87.9)	85 (73.28)
No formal education	100 (52.36)**	73 (38.22)**	107 (56.02)**
Primary education	937 (79.61)	798 (67.80)	867 (73.66)
Secondary Education	399 (94.33)	351 (82.98)	342 (80.85)
Employment status			
Government sector	76 (96.20)	70 (88.6)	57 (72.15)
Housewife	229 (70.68)**	189 (58.3)**	223 (68.83)**
Private Sector	103 (93.64)	91 (82.7)	81 (73.64)
Self employed	872 (84.25)	731 (70.6)	779 (75.27)
Student	36 (94.7)	31 (81.6)	32 (84.2)
Unemployed	232 (72.3)**	212 (66.0)**	229 (71.3)
Family member been infected with COVID-19			
Do not Know	-	26 (33.3)**	38 (48.7)
No	1421 (80.1)**	1181 (69.6)**	1268 (74.7)
Yes	127 (96.2)	117 (88.6)	95 (71.9)
Received vaccine against other diseases			
I do not know		-	
No	254 (65.1)**	194 (49.7)**	247 (63.3)*
Yes	1294 (85.3)	1130 (74.5)	1154 (76.1)
Region			
Arusha	187 (77.9)	135 (56.2)**	133 (55.4)**
Iringa	203 (84.6)	178 (74.2)	197 (82.1)
Kagera	238 (100.0)	233 (97.9)	216 (90.8)
Katavi	152 (65.8)	162 (70.1)	179 (77.5)
Manyara	191 (80.2)	141 (59.2)**	157 (65.9)**
Morogoro	196 (81.7)	175 (72.9)	202 (84.2)
Mtwara	195 (81.2)	133 (55.4)**	178 (74.2)
Tabora	186 (77.5)**	167 (69.6)	139 (57.9)**
COVID-19 is present in our country			
No	67 (36.8)**	52 (28.6)**	86 (47.25)**
Yes	1481 (85.9)	1272 (73.7)	1315 (76.23)
Risk of getting COVID-19 infection			
Low risk	639 (77.5)**	530 (64.3)**	586 (71.12)**
High risk	909 (83.9)	974 (73.3)	815 (75.25)

** P<0.001

Respondents from the general population who were separated/divorced (ARR= 0.8; 95% CI 0.7 to 0.9), had a primary level of education (ARR=0.8; 95% CI 0.7 to 0.9), were self-employed (ARR= 0.8; 95% CI 0.7 to 0.9) and unemployed (ARR= 0.7; 95% CI 0.6 to 0.8) were less likely to report being vaccinated with COVID-19 vaccine. Conversely, those with positive attitude (ARR=1.2; 95% CI 1.1 to 1.5), positive perception (ARR=1.8; 95% CI 1.5 to 2.2), and high knowledge (ARR= 3.0; 95% CI 2.1to 4.4)) on COVID-19 vaccination were more likely to be vaccinated than their counterparts. Respondents with history of chronic diseases (ARR=1.3; 95% CI1.2 to 1.4) and those who had received vaccine against other diseases (ARR=1.2; 95% CI1.0 to1.3) were also likely to be vaccinated. Additionally, respondents from general population who reported that their family members died of COVID-19 were more likely to be vaccinated against the disease (ARR=1.3; 95% CI 1.1to1.4) (Table 8).

**TABLE 8:** Determinants of General Population's Uptake of COVID-19 Vaccine

Variables	CRR (95% CI)	ARR (95% CI)
Marital status		
Married	Ref	Ref
Cohabiting	0.6 (0.4 – 0.9)	0.8 (0.6 – 1.1)
Single	1.0 (0.8 – 1.2)	1.1 (0.9 – 1.3)
Divorced/separated	0.6 (0.5 – 0.8)	0.8 (0.7 – 0.9)
Level of education		
College/University	Ref	Ref
No formal education	0.6 (0.5 – 0.7)	0.9 (0.7 – 1.1)
Primary education	0.6 (0.5 – 0.7)	0.8 (0.7 – 0.9)
Secondary Education	0.8 (0.6 – 0.9)	0.9 (0.8 – 1.1)
Employment status		
Private Sector	Ref	Ref
Government sector	1.4 (0.9 – 1.4)	1.1 (0.9 – 1.3)
Housewife	0.6 (0.5 – 0.8)	0.9 (0.8 – 1.1)
Self employed	0.6 (0.5 – 0.7)	0.8 (0.7 – 0.9)
Student	0.5 (0.4 – 0.6)	0.7 (0.4 – 1.1)
Unemployed	0.5 (0.4 – 0.6)	0.7 (0.6 – 0.8)
History of chronic disease		
No	Ref	Ref
Yes	1.6 (1.4 – 1.8)	1.3 (1.2 – 1.4)
Received vaccine for other diseases		
No	Ref	Ref
Yes	1.6 (1.3 – 1.8)	1.2 (1.0 – 1.3)
Family members died from COVID-19		
No	Ref	Ref
Yes	1.7 (1.5 – 1.9)	1.3 (1.1 – 1.4)
COVID-19 vaccine knowledge		
Low	Ref	Ref
High	5.4 (3.9 – 7.6)	3.0 (2.1 – 4.4)
Perception on COVID-19 vaccine		
Negative	Ref	Ref
Positive	1.6 (1.4 – 1.9)	1.8 (1.5 – 2.2)
Attitude towards COVID-19 vaccine		
Negative	Ref	Ref
Positive	3.2 (2.6 – 3.8)	1.2 (1.1 – 1.5)

## DISCUSSION

Vaccination against COVID-19 stands as a crucial and lifesaving preventive measure against the COVID-19 pandemic.^16–19^ Vaccinating at least 70% or more of the population is imperative for establishing herd immunity, reducing disease severity and mortality, and ultimately halting the epidemic.^17,20,21^ Despite the importance of vaccination, Tanzania's coverage of COVID-19 vaccination remains significantly low.

The 42% uptake of COVID-19 vaccine reported by the current study is higher than 18% reported by the study conducted in 6 regions of the Tanzanian mainland and 2 in Zanzibar between December 2021 and April 2022.^16^ The disparity may be attributed to the timing of the Msuya et al^16^ study, conducted during a period of heightened vaccine hesitancy among the general community members in Tanzania. Additionally, regions which participating in that study were selected based on the vaccine wastage rates, with regions experiencing high vaccine wastage demonstrating lower COVID-19 vaccine uptake. The current study was implemented between July and August, 2022 after the country had expanded its COVID-19 vaccination awareness campaigns and other outreach activities. Furthermore, and the National Vaccine Development Plan (NVDP) underwent revision to ensure that rural populations' access to vaccinations and enhance the capacity of health workers and facilities for vaccine delivery. Nevertheless, the reported COVID-19 vaccination coverage of 42% in this study falls below the global target of 70% and country target of 60%.

The heightened knowledge, and more favourable attitudes and perceptions on COVID-19 vaccination reported in this study may be the results of concerted efforts by the government and other stakeholders to raise community awareness about the importance of accepting the vaccine, in order to curtail the spread of the virus. Prior to these government intervention, people's perception towards COVID-19 vaccines was poor.^22^

Generally, HWs showed a higher likelihood of being vaccinated against COVID-19 compared to the general population, a trend possibly cushioned by their comprehensive knowledge, positive attitude and perception regarding COVID-19 vaccination. This may have offset the differences in uptake of COVID-19 vaccination among HWs belonging to various social, demographics and economic groups.

Numerous studies have reported low uptake of COVID-19 vaccine among specific groups including young individuals, females, those with low perceived risk of infection, low education level, low socio-economic status, and those holding negative attitude towards vaccines.^16,17,21,24–31^ Our study demonstrated that respondents from the general population who were separated/divorced, had reached primary level of education, self-employed or unemployed were less likely to report being vaccinated with COVID-19 vaccine.

Consistent with other studies,^16,17,21, 24-31^ our study confirms the role of social-demographic characteristics, knowledge, attitude and perception in shaping COVID-19 vaccination behaviour. Specifically, general population respondents with better knowledge, positive attitude and perception about COVID-19 vaccination were more likely to be vaccinated than their counterparts.

Contrary to Ahmed et al, who reported no significant difference in vaccine acceptance between medical professionals and the general population,^32^ our study revealed that almost all HWs had comprehensive knowledge, and positive attitude and perception on COVID-19 vaccination which may have increased their likelihood of being vaccinated compared to the general population.

Furthermore, while other studies showed that personal loss due to COVID-19 could deter vaccine acceptance,^32^ our study demonstrated an opposite trend. Individuals who had family member infected with or who died from COVID-19 were more likely to have received the vaccine, potentially motivated by the fear of personal infection or loss.

## CONCLUSION

Uptake of COVID-19 vaccine among the general population was significantly low among individuals with primary level education, those who were self-employed, unemployed, and those who were divorced or separated. Individuals with comprehensive knowledge about COVID-19 vaccination, those with positive attitude and perception towards COVID-19 vaccination, having history of chronic diseases, prior vaccination against other diseases, and having a family member who succumbed to COVID-19 increased the likelihood of COVID-19 vaccine uptake among the general population. Provision of health education and implementation of socio-behavioural communication change interventions are necessary to equip the general population with the appropriate knowledge to transform their negative attitude and perception on COVID-19 vaccination. Moreover, refreshers training courses should be provided to health workers as a substantial proportion exhibited limited knowledge about COVID-19 vaccination.
